# Valuing peer review at Disease Models & Mechanisms

**DOI:** 10.1242/dmm.050717

**Published:** 2024-01-30

**Authors:** E. Elizabeth Patton

**Affiliations:** MRC Human Genetics Unit and CRUK Scotland Centre and Edinburgh Cancer Research, Institute of Genetics and Cancer, Western General Campus, The University of Edinburgh, Edinburgh EH4 2XU, UK

To what extent should peer review be a requirement for publication in Disease Models & Mechanisms (DMM)? Last year, at a meeting of the Board of Directors of The Company of Biologists, this provocative question was raised in the context of an informal discussion about preprint servers, which enable authors to distribute their work prior to peer review. My immediate reaction was that of course we need peer review! But the question has stayed with me and challenged me to understand and articulate exactly how peer review serves authors and readers at DMM, and how we can improve this process.

In recent years, preprint servers have emerged as a mechanism for authors to disseminate their own work directly, without a journal publisher. This can be especially beneficial for work funded by charities and the taxpayer, and for early-career researchers and researchers in low- and middle-income countries, because distribution is immediate, free and not defined by the scope of specific journals or publishers (Sarabipour et al., 2019; as described in an article for University World News by Maina Waruru). Rather than formal peer review, readers can directly comment on the interpretation of the data or analysis, and the authors can choose whether or how to respond. In a recent study, it was reported that 7.3% of preprints in bioRxiv and medRxiv from 2020 had some form of comments and that these were similar to the quality of comments in peer review (Carneiro et al., 2023). Preprint servers, including region-specific servers such as AfricArXiv, can help provide visibility for research from low- and middle-income countries, which might struggle with discoverability and the costs of research publishing (article for University World News by Maina Waruru). However, the sharing of data prior to peer review is not without risk, and there can be a direct harm to the field and to the public when low-quality, flawed or even fraudulent studies are posted on preprint servers, as, for example, during the COVID-19 pandemic (Gopalakrishna, 2021). One solution to this is Review Commons, which provides authors with a refereed preprint that can be posted to bioRxiv, as well as the facilitated transfer of the manuscript and reviews to affiliated journals, including DMM and some of its sister journals.

So, as an independent not-for-profit publisher within a highly competitive landscape, what service do we provide authors when they choose to publish their papers with us? Does peer review provide a necessary part of the scientific process or an unnecessary speedbump to dissemination? Might the new eLife model, which enables authors to choose which, if any revisions they perform, be preferred for our editors and authors? I took these questions to the annual DMM Editor meeting in London this past October.

First, we discussed our experience with peer review. As academic editors at DMM and practicing scientists whose own work undergoes scrutiny, our discussion was shaped by our dual perspective. We have found that, since the pandemic, academics are finding an increase in their workload, and it can take longer to secure appropriate reviewers. At DMM, we aim for three reviewers for each article, but if we have two reviewers who can sufficiently cover the experimental range of the work, we can move forward. We are also noticing that, again since the pandemic, reviewers need more time to complete reviews, and we are generally happy to accommodate this and try to keep the authors informed. This stage is the longest in the process, and we are mindful that although authors are anxious to receive the reviews as soon as possible, the reviewers are good-will volunteers (Aczel et al., 2021). To address concerns about publishing times, The Company of Biologists is discussing innovative publishing models that include rapid review and compensating reviewers. Some of the ways we support and incentivise reviewers at The Company of Biologists' journals are outlined in Box 1. From our surveys and feedback, we know that reviewers are motivated by reviewing for a journal published by a not-for-profit company that invests in science and the scientific community (through our funding of, for example, Travelling Fellowships, the Fund for Innovation in Sustainable Conferencing, Meeting Grants and Workshops).

**Box 1. Supporting our reviewers**

**Cross-referee commenting**

DMM operates cross-referee commenting, wherein we invite referees to comment on the other referee reports prior to editorial decision. The aim of this cross-referee commenting step is to help resolve differences between referees, identify unnecessary or unreasonable requests, or, conversely, highlight valid concerns raised by one referee but overlooked by others.

**Acknowledging our reviewers**

A small gesture perhaps, but each year DMM publishes a list of its peer reviewers (and co-reviewers) from the past year.

**Partnership with Web of Science Reviewer Recognition Service**

DMM's partnership with Web of Science Reviewer Recognition Service (formerly known as Publons) allows reviewers to easily track and verify every review by choosing to add the review to their Publons profile when completing the review submission form.

**The Forest of Biologists**

To acknowledge our reviewers, who help preserve the integrity of the scientific record, we fund the restoration and preservation of ancient woodland within Great Knott Wood in the Lake District National Park, UK. Each time a peer reviewer completes the review process for one of our articles, we dedicate a tree in the ancient woodland to them. Representations of these trees are added to our virtual forest periodically. There will be no association with specific articles to ensure that peer reviewers retain their anonymity. Hear from publisher Claire Moulton and read the Editorial to find out more about The Forest of Biologists.

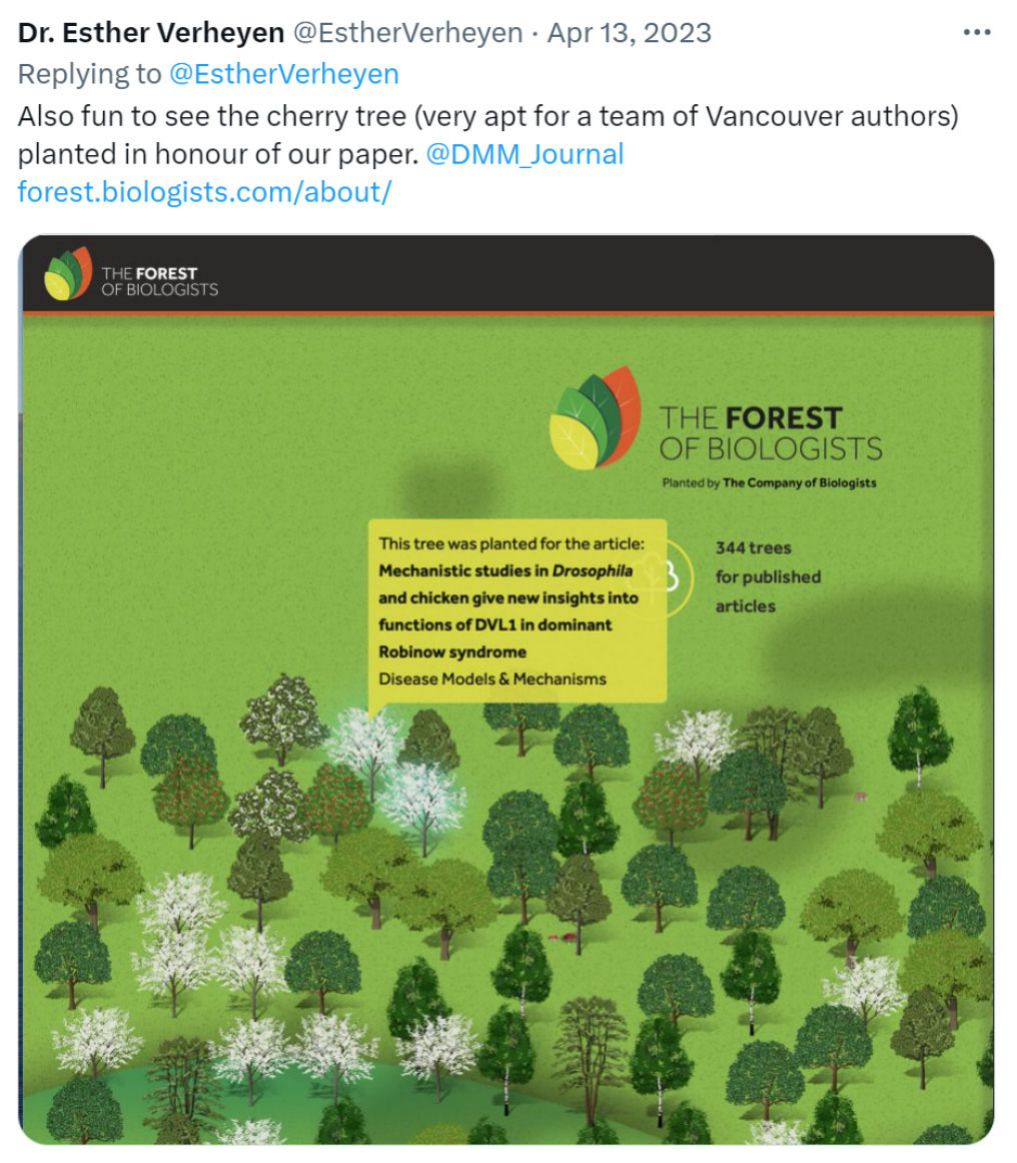

Generally, we agreed that reviewers submitted thoughtful, thorough and considerate critiques of the paper and that, overall, we felt that the revision process improved the scientific rigour and clarity of DMM papers. There is no doubt that it can be an imperfect process, and sometimes the reviewer doesn't get it right, or takes an unnecessarily negative or superficial view. In these cases, it is the role of the editor to communicate to the authors which aspects of the reviews to address and focus their efforts on for revision. At DMM, the Editors and in-house editorial team regularly discuss issues relating to peer review of individual manuscripts to assist in our decision making. And, of course, the authors themselves can respond to the reviewer and explain why some revisions may be helpful, whereas others do not advance the conclusions of the research.

We understand that the peer review process can be subject to bias, including gender, ethnicity, career stage and geography (Caplar et al., 2017; Harris et al., 2017a,b; Helmer et al., 2017; Holst et al., 2022; Smith, 2006; Squazzoni et al., 2021). DMM aims to engage a broad and diverse group of authors, reviewers, Editors, editorial staff, Editorial Board members and readers. The Company of Biologists was a founding signatory of a cross-publisher joint commitment for action on inclusion and diversity in publishing, which is working towards ensuring that we reflect the diversity of the community in our publishing activities, for example, by enabling diversity data to be self-reported by our reviewers and authors. In addition, DMM follows Committee on Publication Ethics (COPE) guidelines for ethical peer review and strives to be aware of and open to new developments and experiments. For example, DMM has recently outlined new policies regarding the use of artificial intelligence in peer review. See Box 2 for some of the new initiatives we are taking at The Company of Biologists regarding peer review.

**Box 2. The future of peer review at The Company of Biologists and DMM**

**Transparent peer review**

Authors who submit to DMM's sister journals Development and Journal of Cell Science can opt to publish ‘peer review history’ files alongside published manuscripts. These include decision letters, referee reports and author point-by-point responses, along with a timeline of the submission and revision process, and the name of the Handling Editor. At this time, these do not include the reviewer names. Confidential comments to the Editor will remain confidential (although we encourage referees to use these only under exceptional circumstances and would prefer all information to be included in the report to the authors), as will comments made through the cross-referee commenting process.

**Peer review badge**

New badges will indicate that papers have undergone rigorous peer review at DMM. We hope that these will be a marker of esteem for authors, and of assurance for readers. This marker is encouraged as good publishing practice to distinguish papers from non-peer-reviewed preprints and is particularly important when communicating science to the public.

**ORCID**

Integration with ORCID will allow reviewers to automatically record their reviewer record with their ORCID account, giving credit where it is due. The profile can then be used in job, visa and grant applications, complete with journal-verified review activities.

One aspect of peer review at DMM that we found very helpful is the cross-referee commenting system that provides a 48-h window for reviewers to comment on each other's reports before an editorial decision is made. Often, having read the other reports, the reviewers will refine their reviews, or even comment where they think other reviewers have misunderstood an experiment or have asked for work beyond the scope of the submission. These comments are incredibly helpful to us editors, as they enable us to distinguish between revisions that truly advance the discoveries in the paper and those that are ‘nice to have’. We can then direct the authors' efforts towards the important revisions.

A flavour of some thoughts about peer review among DMM Editors is given in Box 3. Two topics that are part of ongoing discussion centre around the balance of how much of the review process is accessible to the reader. Two of DMM's sister journals, Development and Journal of Cell Science, operate transparent peer review, which provides the reader with access to the decision letters, referee reports and author point-by-point responses, along with a timeline of the submission and revision process, and the name of the Handling Editor (but not reviewer names) (Holst et al., 2022). Adopting the same approach, to which authors opt in, is still being discussed among the DMM Editor team, with some Editors concerned about exactly how much of the otherwise confidential discussions between authors, reviewers and editors is revealed upon publication. Another area concerns revealing reviewer identity. Although reviewers can sign their names to their review, generally, DMM reviewers are anonymous to the authors and to each other. Some Editors feel that revealing reviewer identity can hold back reviewers from expressing their true opinion about the manuscript to avoid conflict between reviewers, and that it may set up obligations (perceived or otherwise) between authors and reviewers, or even among the reviewers themselves. Others feel that revealing reviewer identity is an important aspect of transparency and allows the authors (and possibly readers, in the case of transparent peer review) to understand the level and area of expertise of the reviewer.

**Box 3. View from the front line – comments from our Editors**

- As an editor, I always learn something new from peer review. I really enjoy seeing people engage with new scientific results, evaluate the science critically, in the best sense of that word, and help improve the manuscript.
- I intentionally include a mixture of scientific perspectives, geography and gender balance when inviting reviewers.
- For me, I see the most important task of the editor as striking the right balance of neutral arbiter, versus ‘managing’ the peer review process. Meaning that I am cautious about intervening between reviewer and author, or overruling a reviewer's requests. At the same time, this is absolutely necessary and appropriate in some cases, especially when it comes to deciding what is ‘reasonable’ to request for author revisions.
- If the importance of anonymous, high-quality peer review is not recognised as requisite for high-quality publications, then trust in the dissemination of scientific findings will be eroded. Already we see the development of a fractured system where it is not clear to all which journals adhere to strong peer review principles and which do not.
- Securing reviewers can be difficult as PIs [principal investigators] have many competing demands. A PI working with an ECR [early-career researcher] to review a manuscript should enable PIs to accept invitations more readily, as the effort in reviewing is reduced and the ECR gains essential experience. [Note: DMM encourages this, as long as there is a genuine mentoring experience, and provides a box to record the co-reviewer name.]
- Quality in peer review requires anonymity, fairness and communication.
- To contribute to diversity in peer review, editorial boards need to be replenished with individuals from diverse backgrounds and geographical locations.
- Peer review can be better rewarded by providing a system wherein reviewers are charged less to publish their manuscript in the journal.
- The review content should be balanced with plusses and negatives, with the negatives couched in an instructional way for how to change the content for the better.
- Peer review is an opportunity to demonstrate what good quality peer review is, and also to educate your mentees and to invite them to be part of the process. This is, after all, really the only way we can perpetuate good peer review into the future.
- There is a perceived lack of diversity or ‘gatekeeping’ inherent in a seemingly elite publishing model. There are a number of things we should work on to encourage diversity in peer review: improving analysis of demographic data and having a clear diversity statement are a start.

We are observing with concern the for-profit Open Access mega journals, which charge a set fee, earn large profits and offer rapid publication, some with a median submission-to-acceptance time within 37 days (Brainard, 2023). Although we firmly believe that diversity in publishing models is best for the scientific community, we are sceptical that many of these journals can provide meaningful peer review at scale. Rigorous peer review is also a critical weapon in the fight against the rise of fraudulent papers generated by ‘paper mills’ (Hackett and Kelly, 2020). Even more concerning, a recent investigation reported in Science shows that paper mills have gone beyond churning out fake papers, and have even infiltrated the publishing process though bribing editors and becoming publishers themselves (Joelving and Retraction, 2024). These practices are eroding trust in specific journals and publishers, with clear impact: over 10,000 papers were retracted in 2023, largely due to concerns about the integrity of the peer review process at Hindawi, a subsidiary of Wiley (Van Noorden, 2023), and 19 Hindawi journals and two MDPI titles were delisted from Web of Science (and ‘lost’ their impact factor) last year (Brainard, 2023). The recently established international group United2Act Against Paper Mills is working with publishers, funders, research bodies and other stakeholders (including Clarivate, the analytics company that runs the Web of Science Master Journal List and calculates the impact factor) and has developed a consensus statement with working groups to address five specific actions to address this problem (https://united2act.org/) (Sanderson, 2024).

One issue that inspires only agreement amongst DMM Editors is the importance of peer review as a marker of quality assurance. In addition, as James Briscoe, Editor-in-Chief of Development, noted, ‘as we all write papers in the knowledge that they will go through peer review, we anticipate criticisms and weaknesses, and in this way, peer review has already influenced studies when they first appear as preprints.’ This professional peer pressure for critical thinking prior to submission to a journal or preprint server improves scientific rigor, and has also been reported to help reduce scientific misconduct (Gopalakrishna, 2021).

Published works at DMM and The Company of Biologists serve as a reliable and permanent record for both the scientific community and the public, which is subsequently built upon for future discovery. We are proud of our peer review at DMM and want readers to know that DMM articles are peer reviewed. To demonstrate this, we are developing a new initiative to have a ‘peer review’ badge, as well as giving the Editor's name, as additional markers of quality beyond the usual metrics of impact and usage. As publishing and peer review continue to evolve, we welcome feedback from authors and reviewers at any time, and authors are sent a survey to complete at time of acceptance or rejection. We aim to continue to be a trusted home for and source of rigorous science.

It is a privilege to handle papers and engage with reviewers at DMM. As DMM Editors, we know how much time peer review and revisions take for both the reviewers and the authors (Aczel et al., 2021). At the start of every year, we thank the reviewers who dedicate their time to contributing thoughtful feedback that truly advances the science and how it is communicated. The names of our 2023 reviewers, including their co-reviewers, are listed in Box 4. We also thank the reviewers of articles transferred to DMM from Review Commons.

**Box 4. Reviewers for Disease Models & Mechanisms 2023**

Amos Abolaji, University of Ibadan, Nigeria

John Abrams, University of Texas Southwestern Medical Center, USA

Helen Abud, Monash University, Australia

Brian Ackley, University of Kansas, USA

Andrew Advani, St. Michael's Hospital, Canada

Johannes Aerts, Leiden University, the Netherlands

Imran Ahmad, Beatson Institute for Cancer Research, UK

Karen J. Aitken, SickKids Research Institute, Canada

Dimuthu Alankarage, Victor Chang Cardiac Research Institute, Australia

Matthew Alexander, University of Alabama at Birmingham, USA

Almundher Al-Maawali, Sultan Qaboos University, Oman

Ahlam Alqahtani, Newcastle University, UK

James Alspaugh, Duke University School of Medicine, USA

James Amatruda, Children's Hospital Los Angeles, USA

Enrique Amaya, University of Manchester, UK

Corina Anastasaki, Washington University in St. Louis, USA

Jimena Andersen, Emory University, USA

Adrienne Antonson, University of Illinois Urbana-Champaign, USA

Jose Aponte, University of Calgary, Canada

Paul Armstrong, University of Leeds, UK

Duchon Arnaud, CNRS, France

Ana Arroba, Instituto de Investigación e Innovación Biomédica de Cádiz, Spain

Sophie Astrof, Rutgers University, USA

Georg Auburger, University Hospital, Germany

Xiaowen Bai, Medical College of Wisconsin, USA

Xiaofei Bai, National Institutes of Health, USA

Sabine Bailly, Laboratoire Biologie du Cancer et de l'Infection, France

Joe Baio, Oregon State University, USA

Adam Bajgar, University of South Bohemia, Czech Republic

Jeroen Bakkers, Hubrecht institute, the Netherlands

Volodymyr Balatskyi, Nencki Institute of Experimental Biology, Poland

Darius Balciunas, Temple University, USA

Rubika Balendra, The Francis Crick Institute, UK

Kathryn Ball, University of Edinburgh, UK

Keir Balla, Chan Zuckerberg Biohub, USA

Erdem Bangi, Florida State University, USA

Scott Baraban, University of California, San Francisco, USA

Andrea Barbuti, University of Milan, Italy

Sami Barmada, University of Michigan, USA

William Barrell, King's College London, UK

Victoria Baxter, Texas Biomedical Research Institute, USA

Bilal Bayazit, Nationwide Children's Hospital, USA

Paola Bellosta, University of Trento, Italy

Anat Ben-Zvi, Ben-Gurion University of the Negev, Israel

Simon Berger, University of Zurich, Switzerland

Jason Berman, Children's Hospital of Eastern Ontario Research Institute/University of Ottawa, Canada

Ambre Bertholet, University of California, Los Angeles, USA

Annabel Berthon, Institut Cochin, France

Cristiano Bertolucci, University of Ferrara, Italy

John Bertram, Monash University, Australia

Sumitha Bharathan, Children's Hospital Los Angeles, USA

Thomas Bird, Beatson Institute for Cancer Research, UK

Judith Birkhoff, Helmholtz Munich, Germany

Lionel Blanc, The Feinstein Institute for Medical Research, USA

Ira Blitz, University of California, Irvine, USA

Robert Bloch, University of Maryland School of Medicine, USA

Karen Blyth, Beatson Institute for Cancer Research, UK

Rolf Bodmer, Sanford Burnham Prebys Medical Discovery Institute, USA

Teresa Bonello, Australian National University, Australia

Eliette Bonnefoy, CNRS, France

Laura Borodinsky, University of California, Davis, USA

Christian Bosselmann, Cleveland Clinic, USA

Luke Boulter, MRC Human Genetics Unit, UK

Chiara Braconi, University of Glasgow, UK

Valerie Brunton, University of Edinburgh, UK

Joseph Brzezinski, University of Colorado, USA

Carol Bult, The Jackson Laboratory, USA

Elisabeth Busch-Nentwich, Queen Mary University of London, UK

Jeffrey Bush, University of California, San Franciso, USA

Ross Cagan, Institute of Cancer Sciences, University of Glasgow, UK

Kirsteen Campbell, Beatson Institute for Cancer Research, UK

Daniel Canarutto, IRCCS Ospedale San Raffaele, Italy

Colette Cann, University of San Francisco, USA

Cathrin Canto, Netherlands Institute for Neuroscience, the Netherlands

Valeria Capra, IRCCS Giannina Gaslini Institute, Italy

Alastair Cardno, University of Leeds, UK

Andrew Carpenter, Oregon State University, USA

Tamara Caspary, Emory University, USA

Pau Castel, NYU Grossman School of Medicine, USA

Deborah Caswell, The Francis Crick Institute, UK

Stephen Cederbaum, University of California, Los Angeles, USA

Silvia Cereghini, Sorbonne Université, Institut Biologie Paris Seine, CNRS UMR7622, France

Mohamed Chahine, Laval University and Cervo Brain Research Centre, Canada

Karen Chang, University of Southern California, USA

Hsiao-Tuan Chao, Baylor College of Medicine, USA

Ching-Hsien Chen, University of California, Davis, USA

Huaiyong Chen, Tianjin University, China

Chung-Ming Chen, National Yang Ming Chiao Tung University, Taiwan

Holly Chen, University of Alabama at Birmingham, USA

Tiantian Chen, University of Florida, USA

Keith Cheng, Pennsylvania State College of Medicine, USA

Philippe Chevalier, University Claude Bernard Lyon 1, France

Brandon Choo, Northeastern University, USA

Wilson Chung, Kent State University, USA

David Church, University of Oxford, UK

Peter Claus, SMATHERIA GmbH, Germany

Martyn Cobourne, King's College London, UK

Robert Coffey, Vanderbilt University, USA

Julien Colombani, University of Copenhagen, Denmark

Matthieu Colpaert, University of Florida, USA

Benjamin Combs, Michigan State University, USA

Simon Conway, Indiana University, USA

Andrew Copp, UCL Great Ormond Street Institute of Child Health, UK

Marianna Cosentino, Sapienza University of Rome, Italy

James Cray, Ohio State University, USA

Mark Cronan, Max Planck Institute for Infection Biology, Germany

Cristiana Cruceanu, Karolinska Institutet, Sweden

Christine Curcio, University of Alabama at Birmingham Heersink School of Medicine, USA

Rodney Dale, Loyola University Chicago, USA

Tatyana Danyukova, University Medical Center Hamburg-Eppendorf, Germany

Ben Davies, The Francis Crick Institute, UK

José de la Pompa, Centro Nacional de Investigaciones Cardiovasculares Carlos III, Spain

Sofia de Oliveira, Albert Einstein College of Medicine, USA

Boel De Paepe, Ghent University Hospital, Belgium

Gonzalo del Monte Nieto, Monash University, Australia

Isabel Del Pino, Instituto Neurociencias Alicante, Spain

Jeroen den Hertog, Hubrecht Institute, the Netherlands

Nicolas Denans, St. Jude Children's Research Hospital, USA

Qing Deng, Purdue University, USA

Marta Derecka, St. Jude Children's Research Hospital, USA

Julianna Determan, Washington University School of Medicine in St. Louis, USA

Pieterjan Dierickx, Max Planck Institute for Heart and Lung Research, Germany

Filomena Digilio, Istituto di Ricerca sugli Ecosistemi Terrestri, UOS Naples-CNR, Italy

Santosh D'Mello, Louisiana State University Shreveport, USA

Leonard Dobens, University of Missouri-Kansas City, USA

Karamjit Singh Dolt, Leiden University Medical Center, the Netherlands

Wolfgang Driever, University of Freiburg, Germany

Carrie Duckworth, University of Liverpool, UK

Philip Dunne, Queen's University Belfast, UK

Agnieszka Dyrda, University of Western Australia, Australia

James Edgar, University of Cambridge, UK

David Eisenstat, University of Alberta, Canada

Masato Enomoto, Kyoto University, Japan

James Ervasti, University of Minnesota, USA

Charlotte Esser, IUF Duesseldorf, Germany

Jeffrey Essner, Iowa State University, USA

Carlos Estella, Centro de Biología Molecular Severo Ochoa - CSIC/UAM, Spain

Diane Fatkin, Victor Chang Cardiac Research Institute, Australia

Annette Feigenbaum, University of California, San Diego, USA

Sarah-Maria Fendt, KU Leuven, Belgium

Yi Feng, University of Edinburgh, UK

Hui Feng, Boston University, USA

Samuele Ferrari, San Raffaele Scientific Institute, Italy

Carlos Ferreira, National Institutes of Health, USA

Maciej Figiel, Institute of Bioorganic Chemistry, Polish Academy of Sciences, Poland

Anthony Firulli, Indiana University School of Medicine, USA

Dustin Flanagan, Monash University, Australia

Bernd Fleischmann, University of Bonn, Germany

Heidi Fuller, Keele University, UK

David Furness, Keele University, UK

Gabriel Galea, UCL Great Ormond Street Institute of Child Health, UK

Rene Galindo, University of Texas Southwestern Medical Center, USA

Daniel Garry, University of Minnesota, USA

Anthony Gavalas, German Center for Diabetes Research/Paul Langerhans Institute Dresden, Germany

Wanzhong Ge, Zhejiang University School of Medicine, China

Matthew Gentry, University of Florida, USA

Maurizio Giustetto, University of Turin, Italy

Jaya Gnana-Prakasam, Saint Louis University School of Medicine, USA

Annie Godwin, University of Portsmouth, UK

Rocco Gogliotti, Loyola University Chicago, USA

Andy Golden, National Institute of Diabetes and Digestive and Kidney Diseases/National Institutes of Health, USA

James Goldenring, Vanderbilt University Medical Center, USA

Mary Goll, University of Georgia, USA

Catia Gomes, Indiana University School of Medicine, USA

Marcus Goncalves, Weill Cornell Medicine, USA

Anai Gonzalez Cordero, Children's Medical Research Institute, Australia

Todd Graham, Vanderbilt University, USA

Stephanie Grainger, Van Andel Institute, USA

Rebecca Green, University of Pittsburgh, USA

Jeremy Green, King's College London, UK

Christopher Gregory, University of Edinburgh, UK

William Grey, University of York, UK

Obi Griffith, Washington University, USA

Brock Grill, University of Washington and Seattle Children's Research Institute, USA

Rosellina Guarascio, UCL Institute of Ophthalmology, UK

Sara Guerreiro, University of Minho, Portugal

Rachel Guest, University of Edinburgh, UK

Liubov Gushchina, Abigail Wexner Research Institute at Nationwide Children's Hospital, USA

Melanie Haffner-Luntzer, University Medical Centre Ulm, Germany

Alex Hajnal, University of Zurich, Switzerland

Martina Hallegger, The Francis Crick Institute and University College London, UK

Ada Hamosh, Johns Hopkins University, USA

Natasha Hanners, University of Texas Southwestern Medical Center, USA

Jens Hansen, Helmholtz Center Munich, Germany

Ross Hardison, The Pennsylvania State University, USA

Hanaa Hariri, Wayne State University, USA

Richard Harland, University of California, Berkeley, USA

Matthew Harris, Harvard Medical School, USA

John Hartman, University of Alabama at Birmingham, USA

Christine Hartmann, Universitätsklinikum Münster, Germany

Tiffany Heanue, The Francis Crick Institute, UK

Christopher Heier, Children's National Hospital, USA

Matthew Hemming, Dana-Farber Cancer Institute, USA

Deborah Henderson, Newcastle University, UK

Gretl Hendrickx, KU Leuven, Belgium

Luis Hernandez-Miranda, Charité – Universitätsmedizin Berlin, Germany

Catherine Hogan, Cardiff University, UK

Peter Hohenstein, Leiden University Medical Center, the Netherlands

Livia Hool, University of Western Australia, Australia

Peter Houweling, Murdoch Children's Research Institute, Australia

Bo Hu, Army Medical University, China

Hu Huang, University of Missouri, USA

Kang-Cheih Huang, Baylor College of Medicine, USA

Saskia Hurst, Max Planck Institute for Infection Biology, Germany

Colin Hutton, The Francis Crick Institute, UK

Robert Hynds, University College London, UK

Tatsushi Igaki, Kyoto University, Japan

Myron Ignatius, University of Texas Health Science Center at San Antonio, USA

Akihiro Ikeda, University of Wisconsin-Madison, USA

Gareth Inman, Beatson Institute for Cancer Research, UK

Evgueni Ivakine, Hospital for Sick Children, Canada

Junichi Iwata, University of Texas Health Science Center at Houston, USA

Rene Jackstadt, German Cancer Research Center (DKFZ), Germany

Tobias Janowitz, Cold Spring Harbor Laboratory, USA

Tatiana Jazedje, University of São Paulo, Brazil

Steve Jean, Université de Sherbrooke, Canada

Matthew Jenny, University of Alabama, USA

Brigid Jensen, Jefferson University, USA

Loydie Jerome-Majewska, McGill University, Canada

Dongyu Jia, Kennesaw State University, USA

Yichang Jia, Tsinghua University, China

Rulang Jiang, Cincinnati Children's Hospital, USA

Erin Jimenez, Johns Hopkins University, USA

Yongfeng Jin, Zhejiang University, China

Katrine Johannesen, Department of Genetics, Denmark

Aaron Johnson, Washington University at St. Louis, USA

Colin Johnson, Oregon State University, USA

Cameron Johnstone, Olivia Newton-John Cancer Research Institute, Australia

Elizabeth Jones, Manchester University/St. Mary's Hospital, UK

Diana Juriloff, University of British Columbia, Canada

Nathalie Jurisch-Yaksi, Norwegian University of Science and Technology, Norway

Monica Justice, Hospital for Sick Children, Canada

Erika Kague, University of Edinburgh, UK

Michael Kahn, Beckman Research Institute of City of Hope, USA

Migle Kalvaityte, Vinius University, Italy

Stephen Kamuli, Yale School of Medicine, USA

Masato Kanemaki, National Institute of Genetics, Japan

Peter Kang, University of Minnesota, USA

Madhuri Kango-Singh, University of Dayton, USA

Phillip Karpowicz, University of Windsor, Canada

Fuyuki Karube, Hokkaido University, Japan

Ajith Karunarathne, Saint Louis University, USA

Douglas B. Kell, University of Liverpool, UK

Justin Kenney, Wayne State University, USA

Scott Kesteven, Victor Chang Cardiac Research Institute, Australia

Mireille Khacho, University of Ottawa, Canada

Kamran Khodakhah, Albert Einstein College of Medicine, USA

Mustafa Khokha, Yale University, USA

Bernard Khor, Benaroya Research Institute, USA

Thomas Kidd, University of Nevada at Reno, USA

Kazu Kikuchi, National Cerebral and Cardiovascular Center, Japan

Sang Hwa Kim, University of Wisconsin-Madison, USA

Junzo Kinoshita, Medicinal Safety Research Laboratories, Daiichi Sankyo, Japan

Alfredo Kirkwood, Johns Hopkins University, USA

David Kirsch, Duke University, USA

Eric Klann, New York University, USA

Nikolai Klymiuk, Technical University of Munich, Germany

David Kohrman, University of Michigan, USA

Takefumi Kondo, RIKEN, Japan

Patryk Konieczny, Adam Mickiewicz University in Poznan, Poland

Maria Kontaridis, Masonic Medical Research Institute, USA

Robert Krauss, Icahn School of Medicine at Mount Sinai, USA

Swathy Krishna, The Ohio State University, USA

Jens Kroll, Heidelberg University, Germany

Etty Kruzel-Davila, Galilee Medical Center, Israel

Jakub Kubis, West Pomeranian University of Technology, Poland

Satu Kuure, University of Helsinki, Finland

Angela Laird, Macquarie University, Australia

Nicole Lake, Yale University, USA

Matthias Lambert, Boston Children's Hospital, USA

Madeline Lancaster, MRC Laboratory of Medical Biology, UK

Karen Lange, University College Dublin, Ireland

Valerie Le Sage, University of Pittsburgh, USA

Maria Ledesma-Colunga, Technische Universität Dresden, Germany

Seung Kyu Lee, National Institute on Aging, USA

Harry Leitch, MRC London Institute of Medical Sciences, UK

Monkol Lek, Yale University, USA

Holger Lerche, University of Tübingen, Germany

Jack Leslie, Newcastle University, UK

Cammie Lesser, Harvard University, USA

Yun Li, University of Toronto, Canada

Vivian Li, The Francis Crick Institute, UK

Zhe Li, Brigham and Women's Hospital, USA

Wei Li, University of Alabama at Birmingham, USA

Chunliang Li, St. Jude Children's Research Hospital, USA

Joseph L. Liang, University of British Columbia, Canada

Heiko Lickert, Helmholtz Zentrum München, German Research Center for Environmental Health, Germany

Graham Lieschke, Australian Regenerative Medicine Institute, Australia

Hui Lim, University of Oklahoma Health Sciences Center, USA

Hongbing Liu, Tulane School of Medicine, USA

Alexander Ljubimov, Cedars-Sinai Medical Center, USA

Samantha Loh, University of Cambridge, UK

Hannah Long, University of Edinburgh, UK

Katie Long, King's College London, UK

Guillermo Lopez-Domenech, University College London, UK

Li Ma, University of Southern California, USA

Laura Machesky, Beatson Institute for Cancer Research, UK

Patricia Maciel, University of Minho, Portugal

Kenneth Maclean, University of Colorado School of Medicine, USA

Bilal Malik, UCL Queen Square Institute of Neurology, UK

Valeria Manara, University of Trento, Italy

Roope Mannikko, University College London, UK

M. Manzini, Rutgers University, USA

Mao Mao, University of California, San Francisco, USA

Ulrika Marklund, Karolinska Institute, Sweden

René Marsano, University of Bari, Italy

David Martinez, Yale School of Medicine, USA

Narcisa Martinez-Quiles, Complutense University, Spain

L. Miguel Martins, University of Cambridge, UK

Marco Massimo, King's College London, UK

Denis Matignon, Institut Supérieur de l'Aéronautique et de l'Espace, France

Lisa Maves, Seattle Children's Research Institute, USA

Margot Mayer-Proschel, University of Rochester, USA

Simon McDade, Queen's University Belfast, UK

Robert McDonald, University of Lethbridge, Canada

Colleen McDowell, University of Wisconsin-Madison, USA

Jacqui McGovern, Queensland University of Technology, Australia

Vedanta Mehta, University of Oxford, UK

Aswin Menke, TNO Triskelion Zeist, the Netherlands

Fjodor Merkuri, University of Massachusetts Lowell, USA

Germana Meroni, University of Trieste, Italy

Gretchen Meyer, Washington University in St. Louis, USA

Mohamad Mikati, Duke University, USA

Marja Mikkola, University of Helsinki, Finland

Rachel Miller, McGovern Medical School, USA

Crispin Miller, Beatson Institute for Cancer Research, UK

Andrew Miller, University of Wisconsin-Madison, USA

Richard Mills, Murdoch Children's Research Institute, Australia

Brian Mitchell, Northwestern University, USA

Nadia Mitchell, Lincoln University, New Zealand

Cecilia Moens, Fred Hutchinson Cancer Research Center, USA

Mervyn Monteiro, University of Maryland, USA

Sally Moody, George Washington University, USA

Ryuji Morizane, Harvard Medical School, USA

Enrico Moro, University of Padova, Italy

Christian Mosimann, University of Colorado School of Medicine, Anschutz Medical Campus, USA

Serge Mostowy, London School of Hygiene and Tropical Medicine, UK

Kevin Myant, University of Edinburgh, UK

Shigekazu Nagata, Osaka University, Japan

Aaron Nagiel, Children's Hospital Los Angeles/University of Southern California, USA

Saidas Nair, University of California, San Francisco, USA

Nawazish Naqvi, Emory University, USA

Salvatore Nesci, Università di Bologna, Italy

Sherylanne Newton, University College London, UK

Johan Neyts, University of Leuven (KU Leuven), Belgium

Teresa Niccoli, University College London, UK

Clévio Nóbrega, University of Algarve, Portugal

Scott Nowak, Kennesaw State University, USA

Wendy Aquino Nunez, University of Kansas, USA

Eseiwi Obaseki, Wayne State University, USA

Lori O'Brien, University of North Carolina at Chapel Hill, USA

Natasha O'Brown, Harvard Medical School, USA

Marcin Osuchowski, Ludwig Boltzmann Institute for Experimental and Clinical Traumatology, Austria

Lisa Ott de Bruin, Leiden University, the Netherlands

Menno Oudhoff, Norwegian University of Science and Technology, Norway

Raghu Padinjat, National Centre for Biological Sciences, India

John Parant, University of Alabama at Birmingham, USA

Nuria Paricio, University of Valencia, Spain

Liz Patton, University of Edinburgh, UK

R. Payne, Indiana University School of Medicine, USA

Nesibe Peker, University of Glasgow, UK

Caroline Pellet-Many, Royal Veterinary College, UK

María Jesús Perugorria, Universidad del País Vasco, Spain

Toby Phesse, Cardiff University, UK

Richard Piercy, Royal Veterinary College, UK

Shubhangi Pingle, Regional Occupational Health Center (S), National Institute of Occupational Health, India

Scott Plafker, Oklahoma Medical Research Foundation, USA

Steve Pollard, University of Edinburgh, UK

Enzo Porrello, Murdoch Children's Research Institute, Australia

Tracey Porter, University of Notre Dame, France

David Pritchard, University of Liverpool, UK

Victor Puelles, University Medical Center Hamburg-Eppendorf, Germany

Paul Quax, Leiden University Medical Center, the Netherlands

Roxana Radu, University of California, Los Angeles, USA

Jill Rafael-Fortney, Ohio State College of Medicine, USA

Shreya Raghavan, Texas A&M University, USA

Catharine Rankin, University of British Columbia, Canada

John Rawls, Duke University School of Medicine, USA

Marina Reichlmeir, University Hospital Frankfurt, Germany

David Reiner, Texas A&M University, USA

Stephen Renshaw, University of Sheffield, UK

Bruno Reversade, King Abdullah University of Science and Technology, Saudi Arabia

David Rice, University of Helsinki, Finland

Karine Rizzoti, The Francis Crick Institute, UK

Liz Robertson, University of Oxford, UK

Carla Robles-Espinoza, Universidad Nacional Autónoma de México, Mexico

Aldo Roccaro, Azienda Socio Sanitaria Territoriale degli Spedali Civili di Brescia, Italy

Christian Rocheleau, Research Institute of the McGill University Health Centre, Canada

Randall Roper, Indiana University Perdue University Indianapolis, USA

Jessica Rosati, Fondazione IRCCS Casa Sollievo della Sofferenza, Italy

Emily Rosowski, Clemson University, USA

Ryan Ross, Rush University Medical Center, USA

Markus A. Rüegg, University of Basel, Switzerland

Avnika Ruparelia, University of Melbourne, Australia

Aimee Ryan, McGill University/Research Institute of the McGill University Health Centre, Canada

Kirsten Sadler Edepli, New York University Abu Dhabi, United Arab Emirates

Takuya Sakaguchi, Cleveland Clinic, USA

Beatriz Salvador, Cardiff University, UK

Federico Sanchez-Quinto, Instituto Nacional de Medicina Genómica, Mexico

Lisa Sandell, University of Louisville, USA

Veronika Sander, University of Auckland, New Zealand

Leslie Sanderson, Erasmus Medical Center, the Netherlands

Simone Sanna-Cherchi, Columbia University, USA

Hiroko Sano, Kurume University, Japan

Celine Santiago, Victor Chang Cardiac Research Institute, Australia

Pamela Santonicola, Institute of Biosciences and BioResources - CNR, Italy

Sumana Sanyal, Sir William Dunn School of Pathology, UK

John-Demian (JD) Sauer, University of Wisconsin-Madison, USA

Katharina Scheibner, Helmholtz Munich, Germany

Barbara Schneider, University of Texas at Arlington, USA

Ben Schumann, The Francis Crick Institute, UK

Jens Schwamborn, Luxembourg Centre for Systems Biomedicine, University of Luxembourg, Luxembourg

John Sedivy, Brown University, USA

Bhuvaneish Selvaraj, University of Edinburgh, UK

Henrik Semb, Helmholtz Zentrum, Germany

Chantelle Sephton, Laval University, Canada

Eduardo Sequerra, Universidade Federal do Rio Grande do Norte, Brazil

Ji Shanming, Institute of Molecular and Cell Biology Strasbourg, France

Jordan Shavit, University of Michigan, USA

Hongying Shen, Yale University, USA

Celia Shiau, University of North Carolina at Chapel Hill, USA

Yuji Shiba, Shinshu University, Japan

Chris Sibley, University of Edinburgh, UK

Roy Sillitoe, Baylor College of Medicine, USA

Rafael Simo, Vall d'Hebron Research Institute, Spain

Marcos Simoes-Costa, Cornell University, USA

Avneesh Singh, University of Maryland School of Medicine, USA

Tulika Singh, University of California, Berkeley, USA

Hazel Sive, Northeastern University, USA

Karl Skorecki, Bar-Ilan University, Israel

Ilya Skovorodkin, University of Oulu, Finland

Andrzej Slominski, University of Alabama at Birmingham, USA

Kelly Smith, University of Melbourne, Australia

Nuri Smith, Graduate Division of Biological and Biomedical Sciences, Emory University, USA

Ian Smyth, Monash University, Australia

Juhoon So, University of Pittsburgh, USA

Charlotte Softley, University Clinic Freiburg, Germany

Masahiro Sonoshita, Institute for Genetic Medicine, Hokkaido University, Japan

Tomokazu Souma, Duke University, USA

André Sousa, University of Wisconsin-Madison, USA

Jason Spence, University of Michigan, USA

Erin Spiller, Heidelberg University, Germany

Knut Stieger, University of Giessen, Germany

Rolf Stottmann, Nationwide Children's Hospital, USA

Helen Strutt, University of Sheffield, UK

Tin Tin Su, University of Colorado at Boulder, USA

Yang Sun, Stanford University, USA

Hoon-Ki Sung, The Hospital for Sick Children, Canada

Amanda Swain, Institute of Cancer Research, UK

Kathleen Sweadner, Massachusetts General Hospital, USA

Sean Sweeney, University of York, UK

Trevor Sweeney, The Pirbright Institute, UK

Francis Szele, University of Oxford, UK

Doaa Taha, The Francis Crick Institute, UK

Krisztina Takacs-Vellai, Eotvos Lorand University, Hungary

Ken Takahashi, Okayama University, Japan

Shin'ichi Takeda, National Institute of Neuroscience, Japan

Jared Talbot, University of Maine, USA

Owen Tamplin, University of Wisconsin-Madison, USA

Ryota Tamura, Keio University School of Medicine, Japan

Mithila Tennakoon, Washington University in St. Louis, USA

Chenglei Tian, Helmholtz Munich, Germany

Randal Tibbetts, University of Wisconsin, USA

Luca Tiberi, University of Trento, Italy

Malte Tiburcy, University Medical Center Göttingen, Germany

Paul Timpson, The Garvan Institute for Medical Research, Australia

Jaafar Tindi, Albert Einstein College of Medicine, USA

Jacques Togo, SickKids Research Institute, Canada

Kristy Townsend, The Ohio State University, USA

Hanh Truong, Memorial Hermann, USA

Suzanne Turner, University of Cambridge, UK

Nigel Turner, Victor Chang Cardiac Research Institute, Australia

Aaron Tward, University of California, San Francisco, USA

Steve Twigg, Oxford University, UK

Victor Tybulewicz, The Francis Crick Institute, UK

Prech Uapinyoying, National Institutes of Health, USA

Cyrille Vaillend, Paris-Saclay Neuroscience Institute, France

Seppo Vainio, Kvantum Institute, Finland

Ainara Vallejo, Instituto Biodonostia, Spain

Bas van Balkom, University Medical Center Utrecht, the Netherlands

Jolanda van Hengel, Ghent Univerisity, Belgium

Sjoerd van Wijk, Goethe University Frankfurt, Germany

Caroline Vance, Kings College London, UK

Johan Vande Voorde, University of Glasgow, UK

Jamie Vandenberg, Victor Chang Cardiac Research Institute, Australia

Jessica Vanslambrouck, Murdoch Children's Research Institute, Australia

Javier Vaquero, Instituto de Investigación Sanitaria Gregorio Marañón, Spain

Neil Vargesson, University of Aberdeen, UK

Elena Vasileva, University of Southern California, USA

Maria Vera, McGill University, Canada

Esther Verheyen, Simon Fraser University, Canada

Alwin Verschueren, IMEC, the Netherlands

Tatyana Vetter, Nationwide Children's Hospital, USA

Mathilakath Vijayan, University of Calgary, Canada

Jeanette Villanueva, Victor Chang Cardiac Research Institute, Australia

Veronique Vitart, University of Edinburgh, UK

Jacy Wagnon, Ohio State University, USA

Peter Walentek, University Freiburg Medical Center, Germany

John Wallingford, University of Texas at Austin, USA

Chenhui Wang, School of Life Science and Technology, ShanghaiTech University, China

Weidong Wang, Laboratory of Genetics, National Institute on Aging, National Institutes of Health, USA

Gordon Warren, Georgia State University, USA

Nicole Weaver, University of Notre Dame Graduate School, USA

Noah Weisleder, Ohio State University, USA

Dominic Wells, Royal Veterinary College, UK

Robert Wheeler, University of Maine, USA

Ann Wheeler, University of Edinburgh, UK

Richard White, University of Oxford, UK

Tanya Whitfield, University of Sheffield, UK

Trevor Williams, University of Colorado, Denver, USA

David Williams, Harvard University, USA

Katherine Wilson, Johns Hopkins University, USA

Rebecca Wingert, University of Notre Dame, USA

Annika Wylie, University of Texas Southwestern Medical Center at Dallas, USA

Hui Xiong, Peking University First Hospital, China

Dongwei Xu, University College London, UK

Hongyuan Yang, University of New South Wales, Australia

Kai-Chun Yang, University of Washington, USA

Chi-Kuang Yao, Academia Sinica, Taiwan

Tsutomu Yasukawa, Nagoya City University Graduate School of Medical Sciences, Japan

Sa Kan Yoo, RIKEN Center for Biosystems Dynamics Research, Japan

H. Joseph Yost, University of Utah, USA

Y. Yu, Roswell Park Cancer Institute, USA

Jane Yu, Victor Chang Cardiac Research Institute, Australia

Kweon Yu, Korea Research Institute of Bioscience and Biotechnology, Republic of Korea

Yizhou Yu, University of Cambridge, UK

Armella Zadoorian, University of New South Wales, Australia

Mayana Zatz, University of São Paulo, Brazil

Michael Zech, Helmholtz Zentrum München, Germany

Jennifer Zhang, Duke University School of Medicine, USA

Tianyi Zhang, National Institutes of Health, USA

Marina Zimmermann, Center for Molecular Neurobiology, University Medical Center Hamburg-Eppendorf, Germany

Irene Zohn, Children's National Health System, USA.
